# The genome sequence of the Hornet Moth, *Sesia apiformis *(Clerck, 1759)

**DOI:** 10.12688/wellcomeopenres.20236.1

**Published:** 2023-10-27

**Authors:** Douglas Boyes, Lindsay Turnbull, University of Oxford and Wytham Woods Genome Acquisition Lab, Darwin Tree of Life Barcoding collective, Wellcome Sanger Institute Tree of Life programme, Wellcome Sanger Institute Scientific Operations: DNA Pipelines collective, Tree of Life Core Informatics collective, Darwin Tree of Life Consortium

**Affiliations:** 1 UK Centre for Ecology & Hydrology, Wallingford, England, UK; 2 Department of Biology, University of Oxford, Oxford, England, UK

**Keywords:** Sesia apiformis, Hornet Moth, genome sequence, chromosomal, Lepidoptera

## Abstract

We present a genome assembly from an individual male *Sesia apiformis* (the Hornet Moth; Arthropoda; Insecta; Lepidoptera; Sesiidae). The genome sequence is 546.8 megabases in span. Most of the assembly is scaffolded into 31 chromosomal pseudomolecules, including the Z sex chromosome. The mitochondrial genome has also been assembled and is 15.4 kilobases in length. Gene annotation of this assembly on Ensembl identified 16,358 protein coding genes.

## Species taxonomy

Eukaryota; Metazoa; Eumetazoa; Bilateria; Protostomia; Ecdysozoa; Panarthropoda; Arthropoda; Mandibulata; Pancrustacea; Hexapoda; Insecta; Dicondylia; Pterygota; Neoptera; Endopterygota; Amphiesmenoptera; Lepidoptera; Glossata; Neolepidoptera; Heteroneura; Ditrysia; Apoditrysia; Sesioidea; Sesiidae; Sesiinae; Sesiini; *Sesia*; *Sesia apiformis* (Clerck, 1759) (NCBI:txid748215).

## Background

*Sesia apiformis* (the Hornet Moth) is a striking member of the clearwing family (Sesiidae) with bold black and yellow stripes on the abdomen. The yellow head and tegulae distinguish it from the Lunar Hornet Moth ( *S. bembeciformis*) which it otherwise closely resembles. Most records are from western and central Europe with a scattering further East into central Russia ( GBIF Secretariat, 2023). The moth has also been introduced into North America, although it is currently confined to the East coast and around the Great Lakes and may be constrained from spreading further because of the presence of the American hornet moth ( *S. tibialis*) which is widespread. In the UK, *S. apiformis* is commonest in the East of England ( NBN Atlas Partnership, 2023), although Nationally Scarce. Adults are on the wing from mid-June to July.

Hornet moths are rarely seen, although unforgettable given their large size and appearance. The larvae feed on the wood of black poplar ( *Populus nigra*) and aspen ( *Populus tremula*), tunnelling below the bark in the lower parts of the trunk and roots ( Arundell & Straw, 2001). There they remain for at least two or even three years until ready to pupate, at which time they create an exit hole, typically of around 1 cm in diameter. The pupa uses spines, or adminicula, to manoeuvre itself close to the hole prior to emergence and exuviae can be found protruding slightly from the holes.

Female moths are larger than males. Once emerged, females remain on the trunk of the tree and produce pheromones to attract a mate. In contrast, the smaller males are quickly active, in the hope of picking up the scent. When a male finds a female, he manoeuvres himself to sit upside down beneath the female while copulation occurs, giving an opportunity for human observation ( Turnbull, 2020). The pheromones have been identified and contain two main chemical components, both of which are necessary to attract males ( Francke *et al.*, 2004).

The hornet moth has been accused of causing serious and significant damage to hybrid black poplar trees in England, but detailed research supports the idea that moths mostly attack trees that are already weakened by drought or human impacts on the water table ( Arundell & Straw, 2001). Further investigations have revealed that poplars showing drought-induced crown dieback can recover well even when moths are present ( Straw *et al.*, 2007). The moth does not, therefore, seem to pose a significant risk to trees in the UK.

The moth also has some claim to cultural significance, lending its name to a biplane: the de Havilland Hornet Moth, the rather less successful successor to the de Havilland Tiger Moth (Jackson, 1987). Only 164 aircraft were produced, although small numbers are still in operation. In addition, *S. apiformis* is one of a handful of UK moths with the ability to elicit strong emotions in those fortunate enough to see it ( Turnbull, 2020). The emergence of this extraordinary insect from the bowels of a tree, often growing in inauspicious locations, if seen, will never be forgotten.

## Genome sequence report

**Figure 1.**
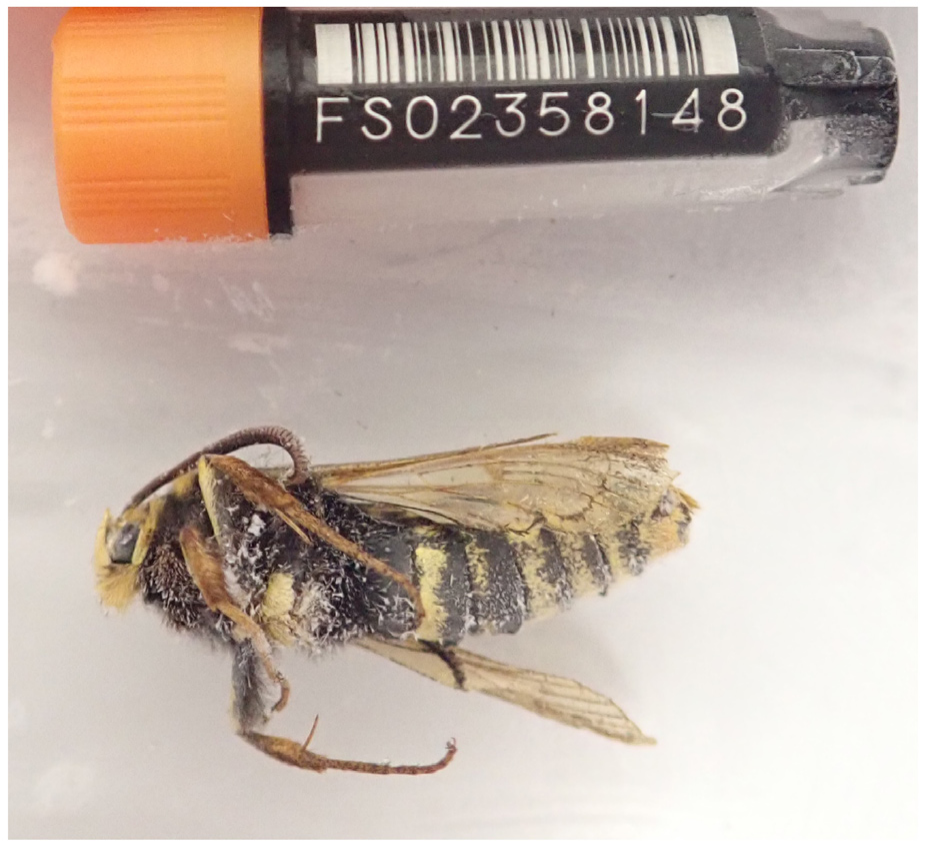
Photograph of the *Sesia apiformis* (ilSesApif2) specimen used for genome sequencing.

The genome was sequenced from one male *Sesia apiformis* ( Figure 1) collected from Wytham Woods, Oxfordshire, UK (51.77, –1.33). A total of 26-fold coverage in Pacific Biosciences single-molecule HiFi long reads and 74-fold coverage in 10X Genomics read clouds were generated. Primary assembly contigs were scaffolded with chromosome conformation Hi-C data. Manual assembly curation corrected 12 missing joins or mis-joins, reducing the scaffold number by 22.22%.

**Figure 2.**
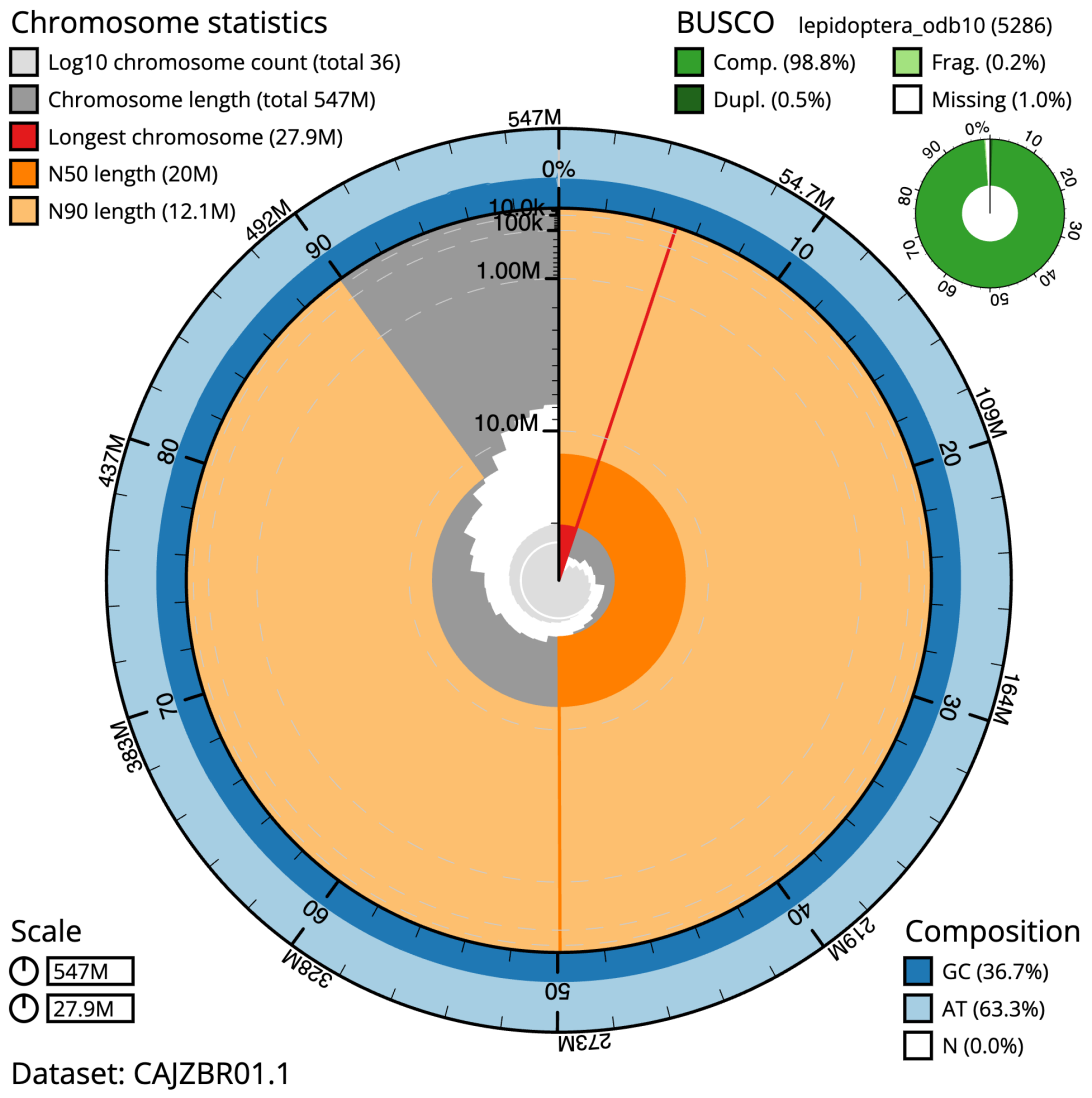
Genome assembly of *Sesia apiformis*, ilSesApif2.1: metrics. The BlobToolKit Snailplot shows N50 metrics and BUSCO gene completeness. The main plot is divided into 1,000 size-ordered bins around the circumference with each bin representing 0.1% of the 546,808,751 bp assembly. The distribution of scaffold lengths is shown in dark grey with the plot radius scaled to the longest scaffold present in the assembly (27,908,545 bp, shown in red). Orange and pale-orange arcs show the N50 and N90 scaffold lengths (19,957,231 and 12,137,843 bp), respectively. The pale grey spiral shows the cumulative scaffold count on a log scale with white scale lines showing successive orders of magnitude. The blue and pale-blue area around the outside of the plot shows the distribution of GC, AT and N percentages in the same bins as the inner plot. A summary of complete, fragmented, duplicated and missing BUSCO genes in the lepidoptera_odb10 set is shown in the top right. An interactive version of this figure is available at https://blobtoolkit.genomehubs.org/view/Sesia%20apiformis/dataset/CAJZBR01.1/snail.

**Figure 3.**
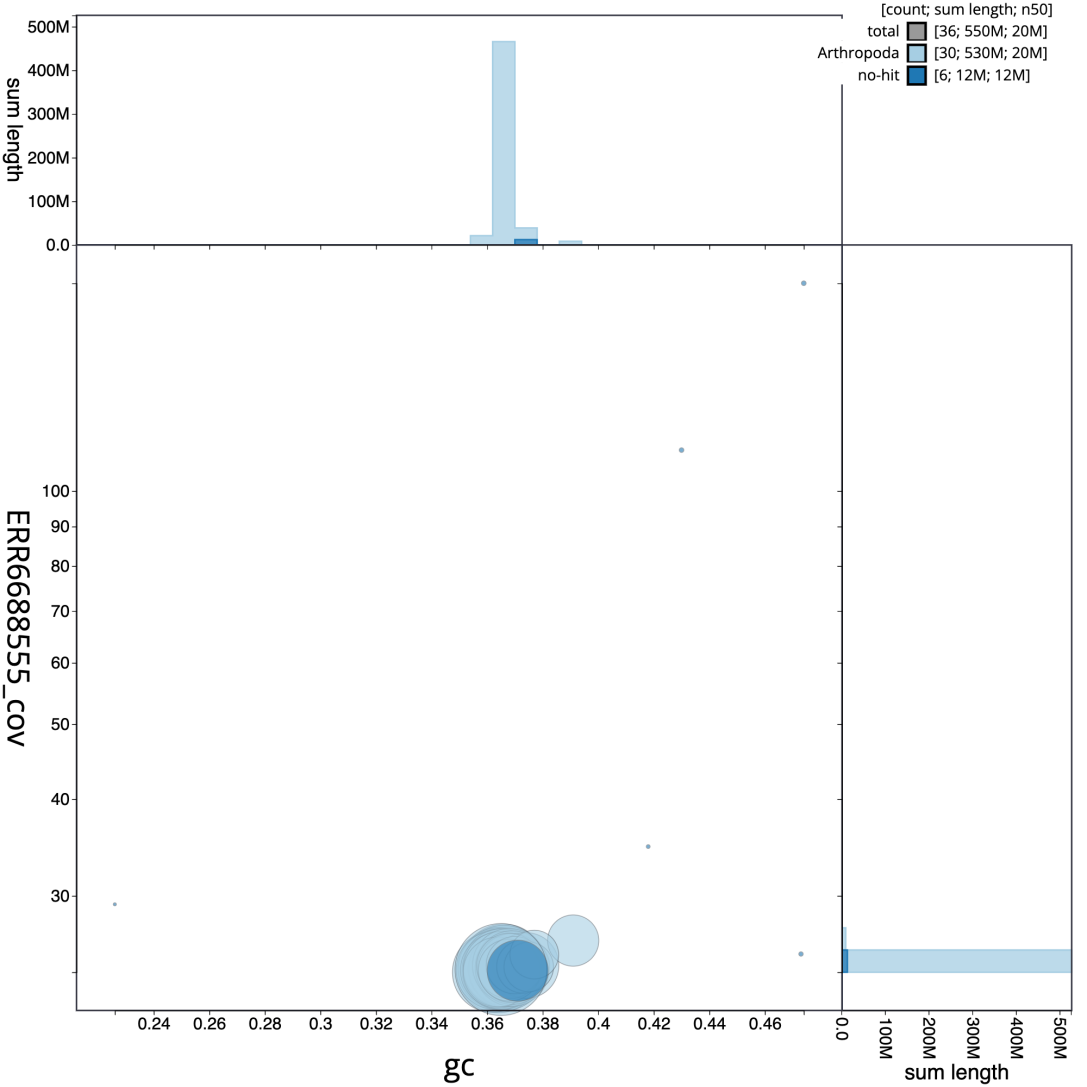
Genome assembly of *Sesia apiformis*, ilSesApif2.1: BlobToolKit GC-coverage plot. Scaffolds are coloured by phylum. Circles are sized in proportion to scaffold length. Histograms show the distribution of scaffold length sum along each axis. An interactive version of this figure is available at https://blobtoolkit.genomehubs.org/view/Sesia%20apiformis/dataset/CAJZBR01.1/blob.

**Figure 4.**
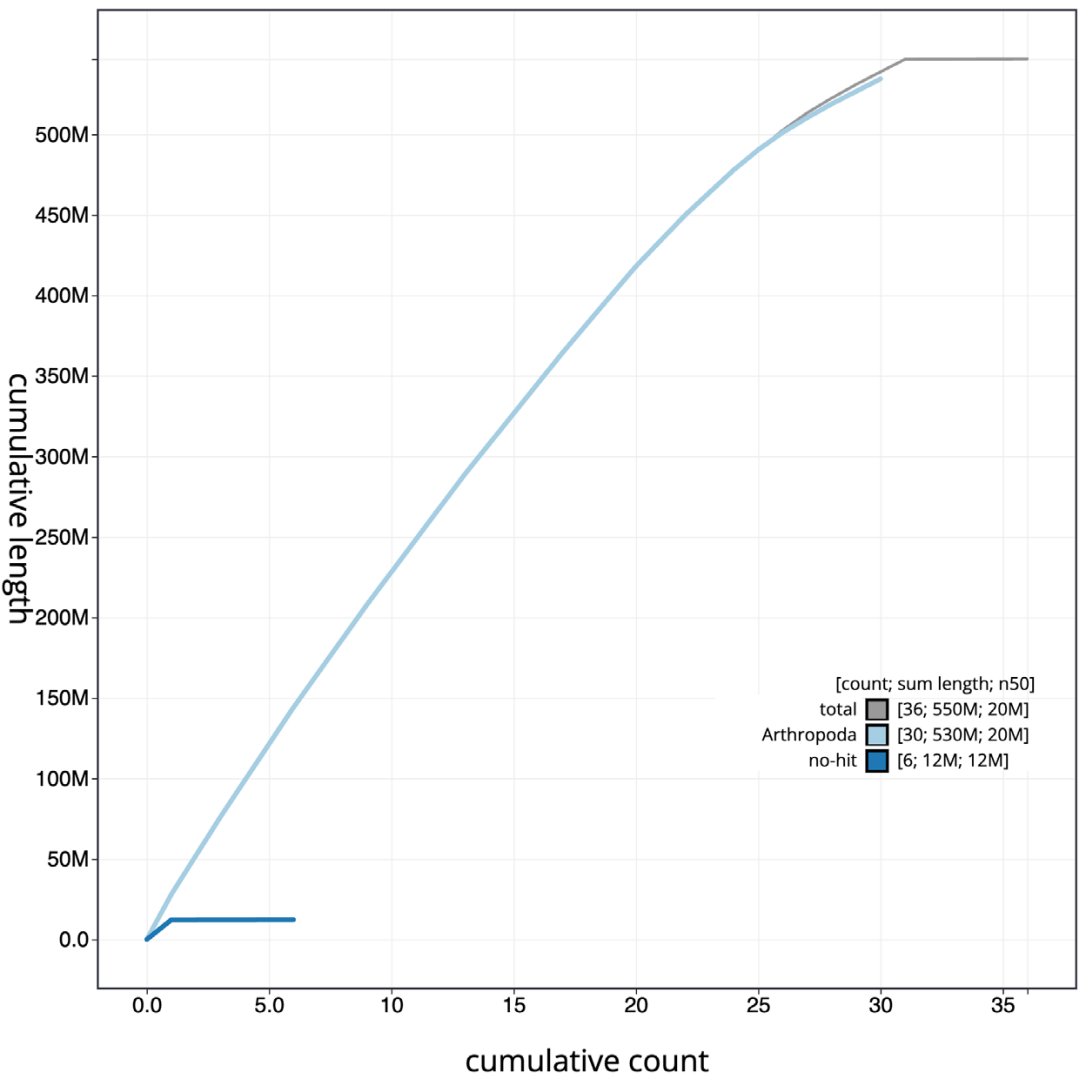
Genome assembly of *Sesia apiformis*, ilSesApif2.1: BlobToolKit cumulative sequence plot. The grey line shows cumulative length for all scaffolds. Coloured lines show cumulative lengths of scaffolds assigned to each phylum using the buscogenes taxrule. An interactive version of this figure is available at https://blobtoolkit.genomehubs.org/view/Sesia%20apiformis/dataset/CAJZBR01.1/cumulative.

**Figure 5.**
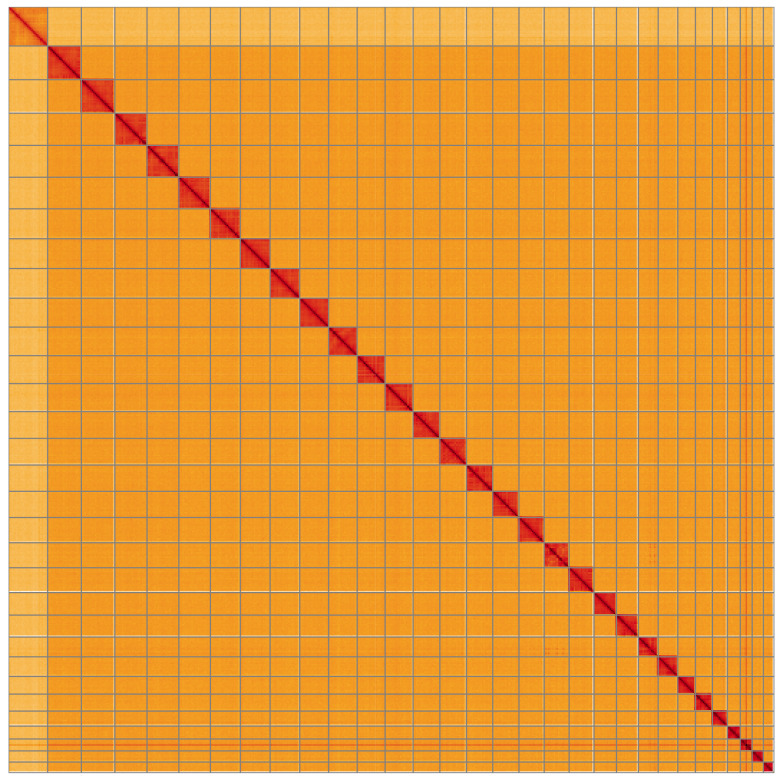
Genome assembly of *Sesia apiformis*, ilSesApif2.1: Hi-C contact map of the ilSesApif2.1 assembly, visualised using HiGlass. Chromosomes are shown in order of size from left to right and top to bottom. An interactive version of this figure may be viewed at https://genome-note-higlass.tol.sanger.ac.uk/l/?d=IkEIGrSCSvGhoDTIyHZaKw.

The final assembly has a total length of 546.8 Mb in 35 sequence scaffolds with a scaffold N50 of 20.0 Mb ( Table 1). A summary of the assembly statistics is shown in Figure 2, while the distribution of assembly scaffolds on GC proportion and coverage is shown in Figure 3. The cumulative assembly plot in Figure 4 shows curves for subsets of scaffolds assigned to different phyla. Most (99.97%) of the assembly sequence was assigned to 31 chromosomal-level scaffolds, representing 30 autosomes and the Z sex chromosome. Chromosome-scale scaffolds confirmed by the Hi-C data are named in order of size ( Figure 5; Table 2). While not fully phased, the assembly deposited is of one haplotype. Contigs corresponding to the second haplotype have also been deposited. The mitochondrial genome was also assembled and can be found as a contig within the multifasta file of the genome submission.

**Table 1.** Genome data for *Sesia apiformis*, ilSesApif2.1.

Project accession data		
Assembly identifier	ilSesApif2.1	
Assembly release date	2021-09-16	
Species	*Sesia apiformis*	
Specimen	ilSesApif2	
NCBI taxonomy ID	748215	
BioProject	PRJEB46325	
BioSample ID	SAMEA7701281	
Isolate information	ilSesApif2 ilSesApif1	
Assembly metrics *		*Benchmark*
Consensus quality (QV)	57.8	*≥ 50*
*k*-mer completeness	99.99%	*≥ 95%*
BUSCO **	C:98.8%[S:98.2%,D:0.5%], F:0.2%,M:1.0%,n:5,286	*C ≥ 95%*
Percentage of assembly mapped to chromosomes	99.97%	*≥ 95%*
Sex chromosomes	Z chromosome	*localised homologous pairs*
Organelles	Mitochondrial genome assembled	*complete single alleles*
Raw data accessions		
PacificBiosciences SEQUEL II	ERR6808007	
10X Genomics Illumina	ERR6688556, ERR6688555, ERR6688558, ERR6688557	
Hi-C Illumina	ERR6688559	
Genome assembly		
Assembly accession	GCA_914767545.1	
*Accession of alternate* *haplotype*	GCA_914767725.1	
Span (Mb)	546.8	
Number of contigs	54	
Contig N50 length (Mb)	20.0	
Number of scaffolds	35	
Scaffold N50 length (Mb)	20.0	
Longest scaffold (Mb)	27.9	
Genome annotation		
Number of protein- coding genes	16,358	
Number of gene transcripts	16,533	

* Assembly metric benchmarks are adapted from column VGP-2020 of “Table 1: Proposed standards and metrics for defining genome assembly quality” from ( Rhie *et al.*, 2021). ** BUSCO scores based on the lepidoptera_odb10 BUSCO set using v5.3.2. C = complete [S = single copy, D = duplicated], F = fragmented, M = missing, n = number of orthologues in comparison. A full set of BUSCO scores is available at https://blobtoolkit.genomehubs.org/view/Sesia%20apiformis/dataset/CAJZBR01.1/busco.

**Table 2.** Chromosomal pseudomolecules in the genome assembly of *Sesia apiformis*, ilSesApif2.

INSDC accession	Chromosome	Length (Mb)	GC%
OU611948.1	1	24.06	36.6
OU611949.1	2	23.9	36.6
OU611950.1	3	23	36.6
OU611951.1	4	22.75	36.6
OU611952.1	5	22.54	36.4
OU611953.1	6	21.31	36.4
OU611954.1	7	21.28	36.2
OU611955.1	8	21.14	36.4
OU611956.1	9	20.64	36.6
OU611957.1	10	20.33	36.3
OU611958.1	11	20.03	36.5
OU611959.1	12	19.96	36.5
OU611960.1	13	19.12	36.5
OU611961.1	14	19.01	36.7
OU611962.1	15	18.75	36.4
OU611963.1	16	18.7	36.5
OU611964.1	17	18.03	36.9
OU611965.1	18	17.84	36.8
OU611966.1	19	17.73	36.9
OU611967.1	20	16.07	36.4
OU611968.1	21	15.62	36.9
OU611969.1	22	14.14	37.4
OU611970.1	23	14.03	36.8
OU611971.1	24	12.55	36.8
OU611972.1	25	12.14	37.1
OU611973.1	26	10.72	37
OU611974.1	27	9.17	37.3
OU611975.1	28	8.52	39.1
OU611976.1	29	7.96	37.5
OU611977.1	30	7.68	37.7
OU611947.1	Z	27.91	36.5
OU611978.1	MT	0.02	22.8

The estimated Quality Value (QV) of the final assembly is 57.8 with *k*-mer completeness of 99.99%, and the assembly has a BUSCO v5.3.2 completeness of 98.8% (single = 98.2%, duplicated = 0.5%), using the lepidoptera_odb10 reference set ( *n* = 5,286).

Metadata for specimens, spectral estimates, sequencing runs, contaminants and pre-curation assembly statistics can be found at https://links.tol.sanger.ac.uk/species/748215.

## Genome annotation report

The *Sesia apiformis* genome assembly (GCA_914767545.1) was annotated using the Ensembl rapid annotation pipeline ( Table 1; https://rapid.ensembl.org/Sesia_apiformis_GCA_914767545.1/Info/Index). The resulting annotation includes 16,533 transcribed mRNAs from 16,358 protein-coding genes.

## Methods

### Sample acquisition and nucleic acid extraction

Specimens of *Sesia apiformis* were collected from Wytham Woods, Oxfordshire (biological vice-county Berkshire), UK (latitude 51.77, longitude –1.33) on 2020-06-23. The specimen was taken from a poplar trunk and then preserved on dry ice. Douglas Boyes (University of Oxford) collected and identified the specimens. One specimen (specimen ID Ox000505, ToLID ilSesApif2) was used for DNA sequencing and another (specimen ID Ox000504, ToLID ilSesApif1) was used for Hi-C data.

DNA was extracted at the Tree of Life laboratory, Wellcome Sanger Institute (WSI). The ilSesApif2 sample was weighed and dissected on dry ice with tissue set aside for Hi-C sequencing. Tissue from the whole organism tissue was disrupted using a Nippi Powermasher fitted with a BioMasher pestle. High molecular weight (HMW) DNA was extracted using the Qiagen MagAttract HMW DNA extraction kit. Low molecular weight DNA was removed from a 20 ng aliquot of extracted DNA using the 0.8X AMpure XP purification kit prior to 10X Chromium sequencing; a minimum of 50 ng DNA was submitted for 10X sequencing. HMW DNA was sheared into an average fragment size of 12–20 kb in a Megaruptor 3 system with speed setting 30. Sheared DNA was purified by solid-phase reversible immobilisation using AMPure PB beads with a 1.8X ratio of beads to sample to remove the shorter fragments and concentrate the DNA sample. The concentration of the sheared and purified DNA was assessed using a Nanodrop spectrophotometer and Qubit Fluorometer and Qubit dsDNA High Sensitivity Assay kit. Fragment size distribution was evaluated by running the sample on the FemtoPulse system.

All protocols used by the Tree of Life laboratory are publicly available on protocols.io ( https://dx.doi.org/10.17504/protocols.io.8epv5xxy6g1b/v1).

### Sequencing

Pacific Biosciences HiFi circular consensus and 10X Genomics read cloud DNA sequencing libraries were constructed according to the manufacturers’ instructions. DNA sequencing was performed by the Scientific Operations core at the WSI on Pacific Biosciences SEQUEL II (HiFi) and Illumina NovaSeq 6000 (10X) instruments. Hi-C data were also generated from head tissue of ilSesApif1 using the Arima2 kit and sequenced on the Illumina NovaSeq 6000 instrument.

### Genome assembly, curation and evaluation

Assembly was carried out with Hifiasm ( Cheng *et al.*, 2021) and haplotypic duplication was identified and removed with purge_dups ( Guan *et al.*, 2020). One round of polishing was performed by aligning 10X Genomics read data to the assembly with Long Ranger ALIGN, calling variants with FreeBayes ( Garrison & Marth, 2012). The assembly was then scaffolded with Hi-C data ( Rao *et al.*, 2014) using SALSA2 ( Ghurye *et al.*, 2019). The assembly was checked for contamination and corrected as described previously ( Howe *et al.*, 2021). Manual curation was performed using HiGlass ( Kerpedjiev *et al.*, 2018) and Pretext ( Harry, 2022). The mitochondrial genome was assembled using MitoHiFi ( Uliano-Silva *et al.*, 2023), which runs MitoFinder ( Allio *et al.*, 2020) or MITOS ( Bernt *et al.*, 2013) and uses these annotations to select the final mitochondrial contig and to ensure the general quality of the sequence.

A Hi-C map for the final assembly was produced using bwa-mem2 ( Vasimuddin *et al.*, 2019) in the Cooler file format ( Abdennur & Mirny, 2020). To assess the assembly metrics, the *k*-mer completeness and QV consensus quality values were calculated in Merqury ( Rhie *et al.*, 2020). This work was done using Nextflow ( Di Tommaso *et al.*, 2017) DSL2 pipelines “sanger-tol/readmapping” ( Surana *et al.*, 2023a) and “sanger-tol/genomenote” ( Surana *et al.*, 2023b). The genome was analysed within the BlobToolKit environment ( Challis *et al.*, 2020) and BUSCO scores ( Manni *et al.*, 2021; Simão *et al.*, 2015) were calculated.

Table 3 contains a list of relevant software tool versions and sources.

**Table 3.** Software tools: versions and sources.

Software tool	Version	Source
BlobToolKit	4.1.7	https://github.com/blobtoolkit/blobtoolkit
BUSCO	5.3.2	https://gitlab.com/ezlab/busco
FreeBayes	1.3.1-17-gaa2ace8	https://github.com/freebayes/freebayes
Hifiasm	0.12	https://github.com/chhylp123/hifiasm
HiGlass	1.11.6	https://github.com/higlass/higlass
Long Ranger ALIGN	2.2.2	https://support.10xgenomics.com/genome-exome/software/pipelines/latest/advanced/other-pipelines
Merqury	MerquryFK	https://github.com/thegenemyers/MERQURY.FK
MitoHiFi	2	https://github.com/marcelauliano/MitoHiFi
PretextView	0.2	https://github.com/wtsi-hpag/PretextView
purge_dups	1.2.3	https://github.com/dfguan/purge_dups
SALSA	2.2	https://github.com/salsa-rs/salsa
sanger-tol/genomenote	v1.0	https://github.com/sanger-tol/genomenote
sanger-tol/readmapping	1.1.0	https://github.com/sanger-tol/readmapping/tree/1.1.0

### Genome annotation

The BRAKER2 pipeline ( Brůna *et al.*, 2021) was used in the default protein mode to generate annotation for the *Sesia apiformis* assembly (GCA_914767545.1) in Ensembl Rapid Release.

### Wellcome Sanger Institute – Legal and Governance

The materials that have contributed to this genome note have been supplied by a Darwin Tree of Life Partner. The submission of materials by a Darwin Tree of Life Partner is subject to the **‘Darwin Tree of Life Project Sampling Code of Practice’**, which can be found in full on the Darwin Tree of Life website here. By agreeing with and signing up to the Sampling Code of Practice, the Darwin Tree of Life Partner agrees they will meet the legal and ethical requirements and standards set out within this document in respect of all samples acquired for, and supplied to, the Darwin Tree of Life Project.

Further, the Wellcome Sanger Institute employs a process whereby due diligence is carried out proportionate to the nature of the materials themselves, and the circumstances under which they have been/are to be collected and provided for use. The purpose of this is to address and mitigate any potential legal and/or ethical implications of receipt and use of the materials as part of the research project, and to ensure that in doing so we align with best practice wherever possible. The overarching areas of consideration are:

•     Ethical review of provenance and sourcing of the material

•     Legality of collection, transfer and use (national and international)

Each transfer of samples is further undertaken according to a Research Collaboration Agreement or Material Transfer Agreement entered into by the Darwin Tree of Life Partner, Genome Research Limited (operating as the Wellcome Sanger Institute), and in some circumstances other Darwin Tree of Life collaborators.

## Data Availability

European Nucleotide Archive: *Sesia apiformis* (hornet moth). Accession number PRJEB46325; https://identifiers.org/ena.embl/PRJEB46325 ( Wellcome Sanger Institute, 2021). The genome sequence is released openly for reuse. The *Sesia apiformis* genome sequencing initiative is part of the Darwin Tree of Life (DToL) project. All raw sequence data and the assembly have been deposited in INSDC databases. Raw data and assembly accession identifiers are reported in Table 1.

## Author information

Members of the University of Oxford and Wytham Woods Genome Acquisition Lab are listed here: https://doi.org/10.5281/zenodo.4789928.

Members of the Darwin Tree of Life Barcoding collective are listed here: https://doi.org/10.5281/zenodo.4893703.

Members of the Wellcome Sanger Institute Tree of Life programme are listed here: https://doi.org/10.5281/zenodo.4783585.

Members of Wellcome Sanger Institute Scientific Operations: DNA Pipelines collective are listed here: https://doi.org/10.5281/zenodo.4790455.

Members of the Tree of Life Core Informatics collective are listed here: https://doi.org/10.5281/zenodo.5013541.

Members of the Darwin Tree of Life Consortium are listed here: https://doi.org/10.5281/zenodo.4783558.
