# Supplementary material for: The genome sequence of the Hornet Moth, Sesia apiformis (Clerck, 1759)
Source: Wellcome Open Res. 2023 Oct 27;8:499. [Version 1] doi: 10.12688/wellcomeopenres.20236.1 (PMC10879760; doi:10.12688/wellcomeopenres.20236.1)
Supplement: Peer review history

# Reviewer report: Reviewer response for version 1

**DOI:** 10.21956/wellcomeopenres.22410.r71563

**Published:** 2024-02-20

**Authors:** Linda Neaves

**Affiliations:** 1 Australian Museum Research Institute, Australian National University, Canberra, Australia

The paper describes the complete genome assembly of the hornet moth ( *Sesia apiformis*) from samples obtained in Wytham Woods Oxfordshire, UK. The assembly was scaffolded into 30 autosomes and the Z sex chromosome. In addition, the mitochondrial genome was also assembled.

 The paper provides a very detailed background information, which includes useful information on the life cycle and impacts of this species on poplars. While interesting I am not sure the number of hornet moth aircraft is relevant.

 Genome sequencing was performed using PacBio HiFi (26x coverage) and 10X Genomics (74x coverage), plus Hi-C data. In the main text it indicates a single male individual from Wytham Wood. However, in the methods two samples are listed, one for the PacBio and 10X and a different individual for the Hi-C. Were both male, and is there an image for the Hi-C sample?

 All the methods are clearly described, along with the resulting data. The final assembly totalled 546.8 Mb with 35 scaffolds and N50 of 20.0 Mb, with a BUSCO completeness rate of 98.8%. This is a very useful piece of work.

Are sufficient details of methods and materials provided to allow replication by others?

Yes

Is the rationale for creating the dataset(s) clearly described?

Yes

Are the datasets clearly presented in a useable and accessible format?

Yes

Are the protocols appropriate and is the work technically sound?

Yes

Reviewer Expertise:

Genomics, evolution, conservation

I confirm that I have read this submission and believe that I have an appropriate level of expertise to confirm that it is of an acceptable scientific standard.

# Reviewer report: Reviewer response for version 1

**DOI:** 10.21956/wellcomeopenres.22410.r71575

**Published:** 2024-01-30

**Authors:** Mohadeseh S Tahami

**Affiliations:** 1 Biological and Environmental Science, University of Jyväskylä (Ringgold ID: 4168), Jyväskylä, Central Finland, Finland

The study presents the complete genome assembly of *Sesia apiformis *moth collected from Oxfordshire, UK. Over 99% of the scaffolded assembly is assigned to 30 autosomes and the Z sex chromosome, plus the complete mitochondrial genome. Detailed taxonomy of the species is given. The authors give a background information on the distribution of the Hornet Moth in Europe and its recent spread into North America, their life cycle, their infestation risk and their cultural impact. Background topics cover a wide scope, although this sentence seems un-necessary: "Only 164 aircraft were produced, although small numbers are still in operation.".

 Genome sequencing was performed using PacBio HiFi technique which yielded 26x coverage, and 10X Genomics technique with x74coverage. Additional Hi-C data was used for scaffolding validation. The final assembly has a total length of 546.8 Mb with 35 scaffolds and N50 of 20.0 Mb. The BUSCO completeness rate is 98.8%, which indicates the reliability of the assembly. The detailed assembling process is very clear and nicely illustrated.

 All in all, it is a neat work and presented in full details.

Are sufficient details of methods and materials provided to allow replication by others?

Yes

Is the rationale for creating the dataset(s) clearly described?

Yes

Are the datasets clearly presented in a useable and accessible format?

Yes

Are the protocols appropriate and is the work technically sound?

Yes

Reviewer Expertise:

taxonomy, phylogeny, genomics, evolution

I confirm that I have read this submission and believe that I have an appropriate level of expertise to confirm that it is of an acceptable scientific standard.
